# Review Application of Nanostructured Black Silicon

**DOI:** 10.1186/s11671-018-2523-4

**Published:** 2018-04-19

**Authors:** Jian Lv, Ting Zhang, Peng Zhang, Yingchun Zhao, Shibin Li

**Affiliations:** 1 State Key Laboratory of Electronic Thin Films and Integrated Devices, School of Optoelectronic Information, University of Electronic Science and Technology of China (UESTC), Chengdu, 610054 People’s Republic of China; 2 Beijing Haidian District Vocational School, 100084 Beijing, People’s Republic of China

**Keywords:** Black silicon, High absorption, SF_6_, Internal quantum efficiency, Application

## Abstract

As a widely used semiconductor material, silicon has been extensively used in many areas, such as photodiode, photodetector, and photovoltaic devices. However, the high surface reflectance and large bandgap of traditional bulk silicon restrict the full use of the spectrum. To solve this problem, many methods have been developed. Among them, the surface nanostructured silicon, namely black silicon, is the most efficient and widely used. Due to its high absorption in the wide range from UV-visible to infrared, black silicon is very attractive for using as sensitive layer of photodiodes, photodetector, solar cells, field emission, luminescence, and other photoelectric devices. Intensive study has been performed to understand the enhanced absorption of black silicon as well as the response extended to infrared spectrum range. In this paper, the application of black silicon is systematically reviewed. The limitations and challenges of black silicon material are also discussed. This article will provide a meaningful introduction to black silicon and its unique properties.

## Background

The high reflectance of traditional silicon, which is higher than 40%, severely limits the applications of silicon-based photon sensitive devices. The large bandgap of 1.07 eV limits the useful wavelength range spectrum of bulk silicon, especially when the wavelength is above 1.1 μm. Moreover, the high reflectance across the electromagnetic spectrum seriously affects the efficiency and sensitivity of optoelectronic devices based on silicon [1]. Black silicon has been studied since 1995, when the micro-structured silicon was fabricated by reactive ion etching (RIE) with high depth-to-width ratio [2]. In the presence of gas atmosphere, silicon with spiked surface has a strong light absorption due to the light-trapping effect: the surface turns deep black and covers with micro-nanospikes after laser irradiation process has been finished, hence namely black silicon [3]. E. Mazur reported that silicon surfaces with arrays of sharp conical spikes and silicon nanoparticles possess higher absorptance in infrared wavelength range when irradiated with 500 femtosecond (fs) laser pulses in SF_6_ [4]. This phenomenon can be ascribed to the sulfur-doping effect in silicon.

With high absorptance in visible and infrared wavelengths, black silicon can be used in visible and infrared photodetectors, solar cells, night vision cameras, and near-infrared (near-IR) avalanche photodiode (APD). Compared to the traditional silicon, the energy band structures of black silicon has been changed, which is beneficial to be used as photoluminescence. As black silicon fabricated with fs lasers is covered with sharp conical micro-spikes arrays, it can be used as field emitters further.

Besides silicon materials, some other semiconductors, for instance indium gallium arsenide and germanium, are always used for near-infrared detection in commercial market. However, these commercial photodetectors show some shortcomings, such as expensive material cost, large noise characteristics, and poor integration with the present silicon-based electronic process. During these years, the scientists are always devoting themselves to finding efficient methods to improve the responsivity of traditional silicon materials [5–8].

## Absorptance Enhanced in Black Silicon

It has been demonstrated that the absorptance of black silicon is enhanced owing to the light-trapping effect of surface morphology and energy level of dopants. In the irradiating process, the parameters of laser pulse, including spot size, pulse number and density, and scanning parameters are crucial to the form of sharp conical micro-spike arrays in substrate surface. While the micro-textured surface greatly decreases the reflection, the absorption in the range from 1100 to 2500 nm is also enhanced due to the doping of chalcogen elements. Both the energy levels of dopants and structural defects would create more intermediate states to enhance the sub-bandgap absorption of silicon. However, the laser irradiation will damage the black silicon surface, resulting in inactive electronic properties. Post-annealing treatment is often used to reduce and repair the damage of structural defects, which aims to improve the carrier mobility without an evident change on silicon surface. The annealing temperature and time should be controlled well because a low annealing temperature would not reduce defect efficiently versus a high-temperature anneal would decrease the below-bandgap absorption of micro-textured silicon significantly. As shown below, it is observed that the absorptance above 1100 nm decreases with the increasing of annealing time under the same annealing conditions. The reduced absorption in the infrared wavelength range depends on the dopant diffusion. It is clear that the samples doped by sulfur element show the largest decrease in infrared absorption, followed by selenium-doped samples and tellurium-doped samples, respectively. Furthermore, the absorption at 1550 nm strongly increases with the increasing number of fs laser pulses.

**Fig. 1:**
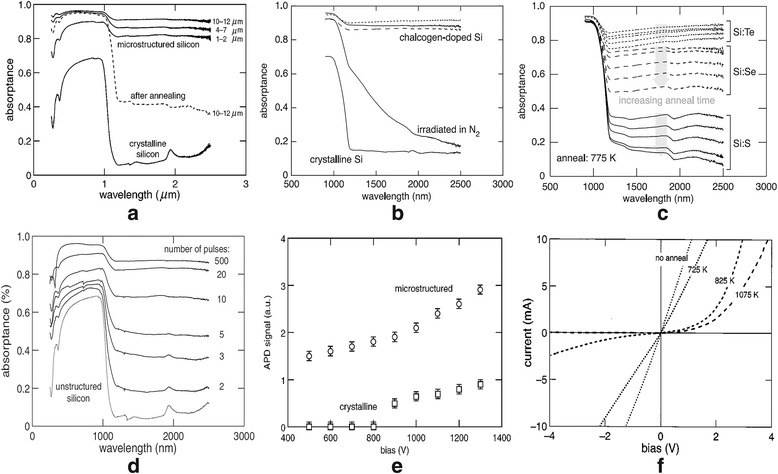
**a** Absorptance of micro-structured and unstructured silicon samples. **b** Absorptance spectra for black silicon samples fabricated under different atmosphere of sulfur hexafluoride (*solid line*), selenium (*dashed line*), tellurium (*dotted line*), and nitrogen gas (*solid line*) [7]. **c** Absorptance spectra of black silicon samples doped with S, Se, and Te ions after thermal annealing at 775 K for different time (from *bottom* to *top*: 24 h, 6 h, 100 min, 30 min, 10 min) [7]. **d** The absorptance of micro-structured black silicon at 1550 nm with respect to the number of laser pulses used in the irradiation processing [8]. **e** Photocurrent curves of the micro-structured and traditional silicon-based avalanche photodiodes (APDs) under a light source of 1.310 μm. **f** I–V curves with different annealing temperature

C. Wu measured the absorptance of crystalline silicon and black silicon before and after annealing shown in Fig. 1a [1]. Brian R. Tull and co-workers modified the boron-doped Si (100) wafers by pre-coating sulfur, selenium, and tellurium powders, respectively, and then used fs laser to irradiate silicon wafers to form the supersaturated concentrations [9]. The obtained absorptance spectra before and after annealing are shown in Fig. 1b, c. It is known that only black silicon doped with chalcogen exhibits high absorption between 1100 and 2500 nm. Brian R. Tull reported that the high concentration of chalcogen dopants in the nanometer-sized grains of the polycrystalline surface layer resulted in the high absorptance near the infrared wavelength [9]. The result is ascribed to the deep level donors created by chalcogen elements in the bandgap of silicon. They provide this explanation by supposing a simple diffusion model: the decrease in absorptance depends on the fraction of dissolved dopants. Upon annealing, these dopants diffuse from the nanometer-sized grains to the grain boundaries of surface layer. The diffusion would reduce the number of donor impurity levels which are cooperated in the bandgap of silicon, thus reducing the infrared absorption.

After thermal annealing, the decrease in the infrared absorptance of silicon with supersaturated chalcogen doping most likely is due to the dopant diffusion. Other mechanisms, such as clustered precipitation of dopant particles within the grains, may lead to a deactivation of infrared absorption to some extent [9]. Black silicon possesses unique optical and electronic properties that are not found in traditional bulk silicon, making it an ideal candidate material for photovoltaic devices.

## Application of Black Silicon

### Photodiodes

Black silicon can be used in traditional junction photodetector architecture. The measured quantum efficiency near the infrared wavelength spectrum is over 10× incumbent than traditional silicon photodetectors, and the former does not exist any significant degradation in terms of noise and other parameters for detectors. With high absorptance in wideband optical spectrum, black silicon photodiodes with high responsivity have been realized by several groups [1, 10–13].

**Fig. 2:**
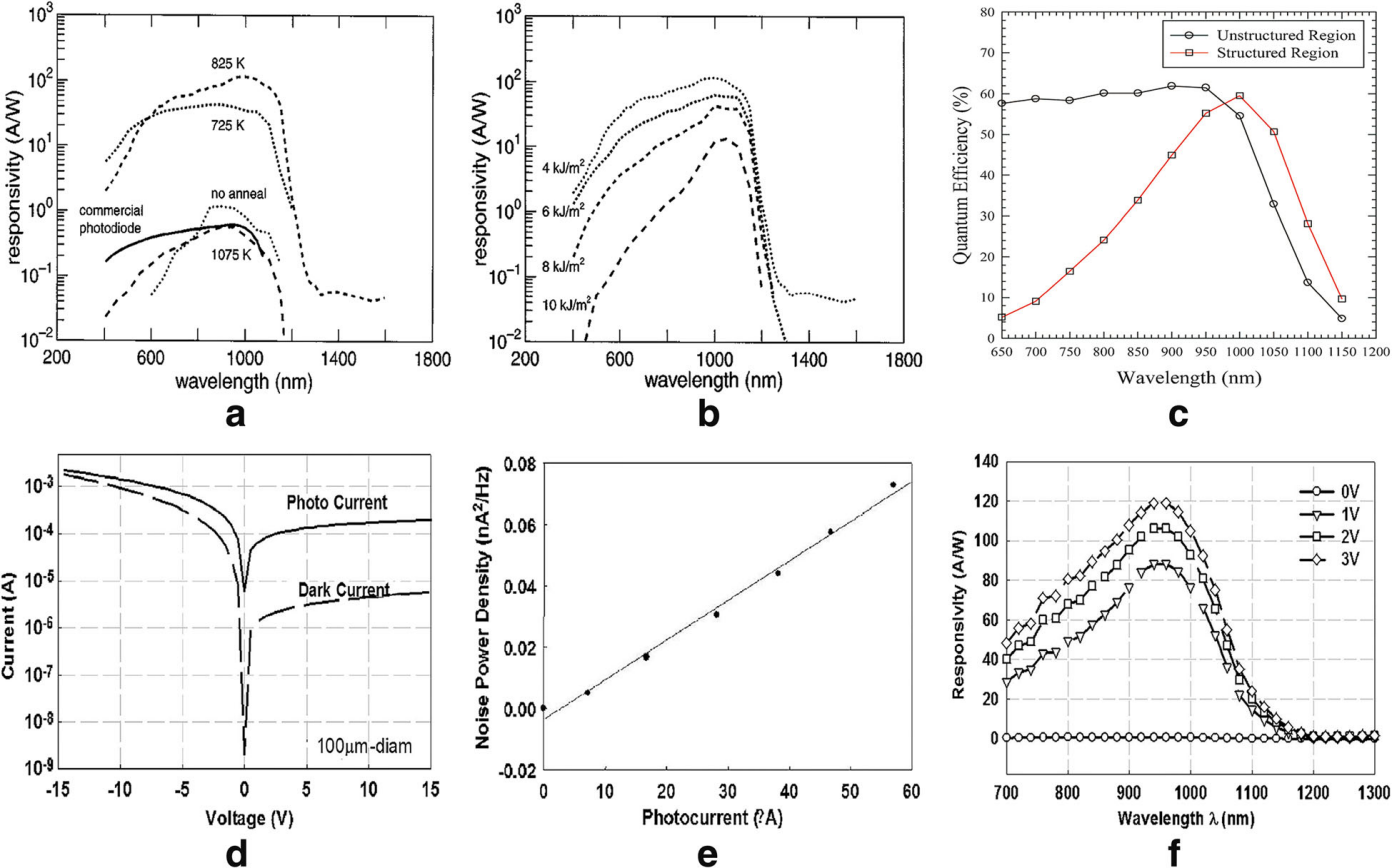
**a** Photoresponsivity with different annealing temperature for each sample is 30 min. **b** Photoresponsivity with different laser fluence. **c** Quantum efficiency depends on the wavelength for the APD that includes the micro-structured and unstructured regions. **d** The current-voltage characteristics of a 100-μm-diameter micro-structured black silicon photodetector [12]. **e** Current noise power density versus photocurrent under applied reverse bias voltage of 3 V. **f** Responsivity for a 250-μm-diameter black silicon device under applied reverse bias of 0, 1, 2, and 3 V [12]

C. Wu fabricated a micro-structured APD with black silicon, which is produced by irradiating a silicon wafer of (111) orientation with a fs laser at 800 nm center wavelength and 100 pulses in SF_6_ [1]. As shown in Fig. 1e, under a bias of 900 V or larger, the production of photocarriers generated from the micro-structured region is at least three times than that from the unstructured region both at 1.064 and 1.310 μm. By using fs laser irradiation in an atmosphere of sulfur-containing, James E. Carey fabricated the high responsivity silicon-based photodiodes for detecting the visible and near-infrared light signal [11]. The photocurrent and responsivity performances of photodiodes strongly depend on the processing conditions, such as substrate dopants, laser fluence, thermal annealing time, and temperature. The measured results are shown in Fig. 1f and Fig. 2a, b.

Optimized black silicon samples exhibit a high responsivity that are almost two and five orders of magnitude higher than that of the commercial silicon photodiodes in the visible and near-infrared wavelength. By using optimized laser parameter, R. Torresa modified the front side of black silicon samples and created the 3D p^+^ junction by using Plasma Immersion Ion Implantation technique to achieve boron implanting [12]. Compared to the non-texturized surface area, it has been demonstrated that the texturized devices show a 57% increase of photocurrent. The traditional silicon PIN photodiodes show a poor absorbing ability to the light above 1.1 μm. Therefore, they cannot be used to detect the two primary telecommunication wavelengths, 1.3 and 1.55 μm. According to Aoife M. Moloney, it was established that an excess increase of 50% in responsivity performance existed in the black silicon surface at 1.1 μm or long wavelengths [13]. Meanwhile, the threshold voltage of black silicon photodiodes is lower than that of the standard silicon-based diodes. The existence of a second photodiode junction formed between the black silicon surface and the silicon substrate made a main contribution to the lower threshold voltage.

Furthermore, Richard A. Myers reported the laser micro-structuring of silicon-based APDs and APD arrays [5]. A series of pre-structured fabrication process, including deep diffusion of boron in a high-temperature diffusion furnace, were used to obtain a 50~ 60 μm p-n junction below the final ~ 250-μm-thick device structure. After annealing, the responsivity of pre-structured device is two to three times higher than the unstructured silicon-based APDs at near-infrared wavelengths. Furthermore, there is no degradation observed from other performance characteristics. They also demonstrated that the increased responsivity at near-infrared wavelengths could be owed to the atmosphere (best in SF_6_) and annealing. But the reduction of quantum efficiency (QE), especially at wavelengths below 900 nm, might be alleviated with additional high temperature annealing, as shown in Fig. 2c.

P. Agarwal et al. demonstrated a highly reproducible embedded silicon nanowire p-n junction diodes, which are fabricated by a fully VLSI compatible etching technology to achieve the diameters below 30 nm [14]. Applied at reverse bias, the heterojunction diodes show a strong relationship between the diameter and breakdown voltage, which maybe results from the surrounding dielectric influence, as shown in Figs. 5b, c.

### Photodetectors

The widely use of silicon in the semiconductor industries drives an extensively interest in improving the responsivity of silicon-based photodetectors in the infrared region. Black silicon allows us to fabricate photodetector devices based on silicon for both the visible and near-infrared wavelengths owing to the high absorption in the range from 250 to 2500 nm [15]. The spectral responsivity for some black silicon devices is nearly ten times greater than that of commercial PIN photodiodes based on silicon materials as used in the visible light.

The responsivity of black silicon detectors has been investigated by several teams with different factors, including annealing temperature, dopants, and background gases. J. E. Carey fabricated silicon-based photodiodes using fs laser-irradiated black silicon [16]. The sensitivity of black silicon detector is ten times than that of commercial PIN photodiodes based on silicon at visible and 1650 nm wavelengths. According to Richard A. Myers, the responsivities of micro-structured silicon APD detectors that were annealed under different conditions were enhanced at near-IR wavelengths [5]. With different background gases, the results demonstrated that black silicon processed in sulfur atmosphere showed the highest possible QE after annealing. It is also demonstrated that the enhanced responsivities of micro-structured APD detectors at long wavelength result from the improved absorption and show nothing to do with the additional energy bands created during laser processing.

As the increasing of total absorption, the decrease of response to short-wavelength radiation was observed in detector, indicating that most of the charge carriers were collected from the deeper area but not from the near-surface region. Post-processed with thermal annealing, the QEs of fabricated APD arrays at 1064 nm were obtained as high as 58% without any degradation of noise, gain, or other electric performances. Also, these experimental results demonstrated that the increased absorption at near-IR made a main contribution to the improved collection of charge carriers.

With fs laser-modified silicon in SF_6_ gas, the photodetectors measured at 3 V bias exhibited high photoresponse of 92 A/W at 850 nm and 119 A/W at 960 nm, respectively [17]. The micro-structured silicon photodetectors still showed strong photoresponse even the wavelengths are longer than 1.1 μm. The photoresponse of these detectors could be explained by a generation-recombination gain mechanism. The gain calculated from the measured results of noise current density was approximately 1200 at 3 V bias. The results of Hall measurements of the surface layer demonstrated electron concentration of micro-structured region was higher than that of substrate, and the electron mobility was on the order of 100 cm^2^ V^− 1^ s^−1^, as shown in Fig. 2d. According to Fig. 2d, at the reverse bias voltages of 1 and 3 V, the dark currents were 1.3 and 2.3 μA for a 100-μm-diameter device, respectively. The values were one order of magnitude lower than the dark current measured at forward-bias under the same voltages. While the photocurrent is increasing, the noise power density linearly increases, as shown in Fig. 2e [17].

**Fig. 3:**
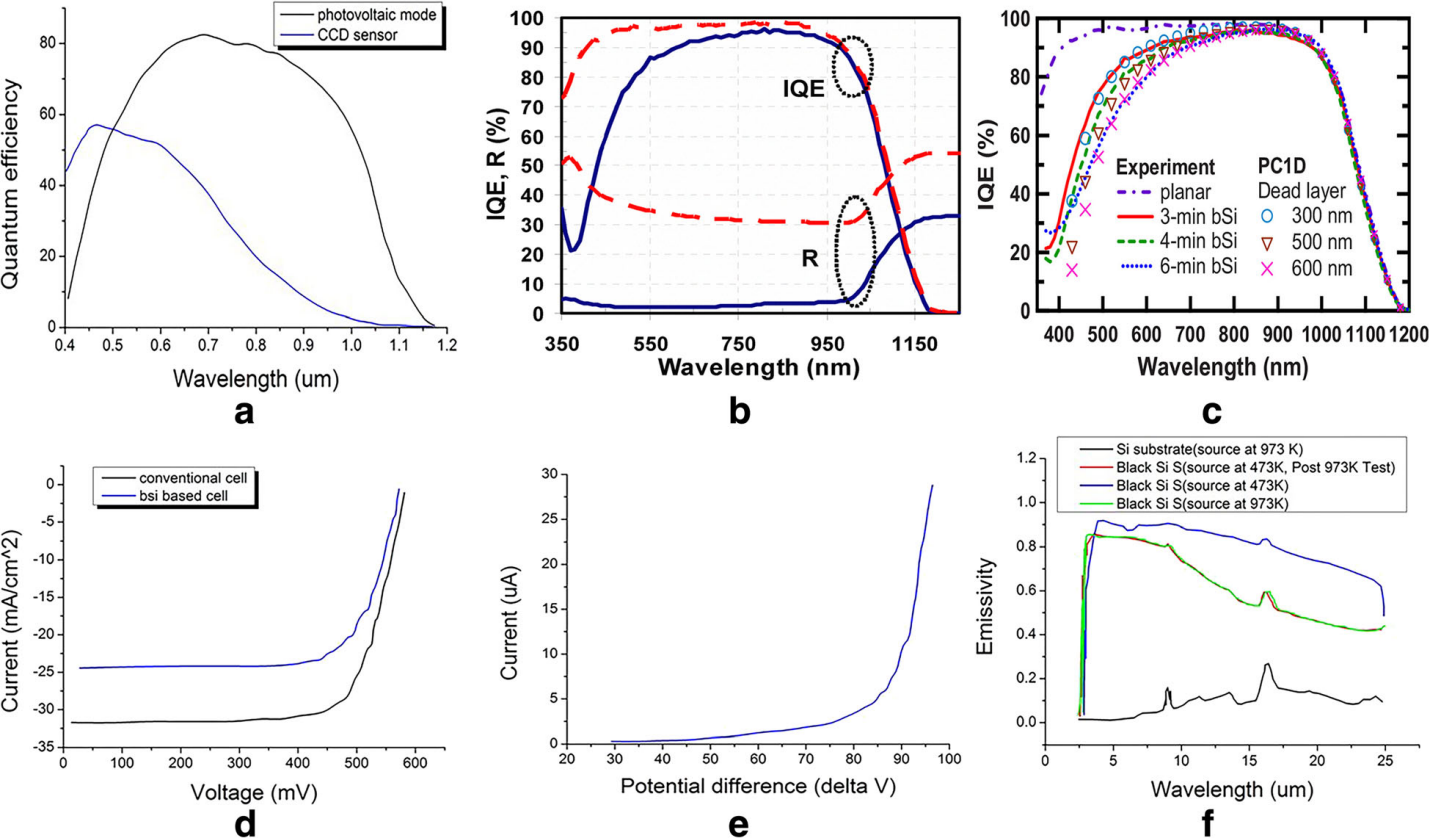
**a** EQE performance for a black silicon photodiode (red) measured in photovoltaic mode and the commercial CCD imaging sensor (blue) [17]. **b** IQE and **c** R measurements for planar silicon-based solar cells and black silicon solar cells. **d** The current-voltage curves of the conventional solar cell and black silicon solar cell made from the SiNW arrays [23]. **e** Current-voltage performances for varied potential differences. Here the spacing of anode-cathode is 20 μm [36]. **f** Emissivity versus wavelength given different blackbody source temperatures [37]

Figure 2f shows the responsivity versus wavelength from 0.60 to 1.30 μm at 0, 1, 2, and 3 V reverse bias [17]. It is clear that the responsivity of black silicon changes with wavelength as single hump, as well as the QE with wavelength (shown in Fig. 3a [18]). M. U. Pralle reported that SiOnyx, Inc., has exploited a novel silicon processing technology for CMOS sensors [18]. The technique would extend spectral sensitivity of traditional silicon-based detectors into the near/shortwave-infrared (NIR/SWIR), thus providing an exciting performance for digital night vision capability. The QE of thin layer is as 10 times as that of incumbent imaging sensors when the spectral sensitivity was measured from 400 to 1200 nm. In the black silicon CMOS, the quantum efficiency at 940 nm is 68%, the dark current at bias voltage of 10 mV is 140 pA/cm^2^, and the response time is 10 ns.

The chalcogen in ambient gas is implanted and incorporated into the silicon surface formed in large concentrations, which influences the photoresponsivity efficiently [18]. In the case of SF_6_, incorporating of sulfur donor is immensely important to achieve the high photoresponsivity. It has been found that the devices with selenium and tellurium incorporation also get the high photo-responsivity. However, other gases such as air, nitrogen, and hydrogen are implanted into the silicon surface, and the devices show a poor responsivity.

James E. Carey reported the application of black silicon in IR sensors [19]. Black silicon exhibits an efficient and high photoconductive gain at room temperature, with responsivities up to more than 100 A/W at NIR. It cannot only dramatically reduce the reflectance of silicon to allow much light to be absorbed at NIR and SWIR region, but also make detectors show high photoresponse from 1000 to 1200 nm. And the responsivity is 100 times higher than that of commercial germanium or InGaAs detectors. M. G. Tanner et al. fabricated the packaged NbTiN SNSPDs (superconducting nanowire single-photon detectors) based on oxidized silicon substrates under the working wavelengths ranging from 830 to 1700 nm [20]. This optical architecture could be optimized for detecting alternative important wavelength signal, such as 1550 nm.

### Solar Cells

The unique light-trapping effect of micro-textured surface morphology greatly enhances the visible absorption of silicon, making it well used in Vis-NIR photodetection, as well as solar cells. By using fs laser without a corrosive gas (under vacuum), M. Halbwax prepared micro- and nanostructured silicon for photovoltaic cells with different nanotexturization methods [21]. And the results demonstrate that the photocurrent owes an increase of ~ 30% in the laser-modified areas. In this study, a photovoltaic structure is made by using a fs laser to illuminate a silicon wafer to achieve locally nanostructured forest (squares of 1 mm^2^). After the laser structuring, the samples have been implanted by boron element using Plasma Immersion Technique (PULSION tool, developed by IBS) to form a p-n junction, followed by rapid thermal annealing (RTA) processing. The absorption of spike structured silicon wafer reaches 94%, which is much higher than that of other structures such as penguin-like, pillars and pyramids, even the absorption of a flat silicon wafer is only 65%. An average photocurrent of the unmodified silicon sample is on the order of 15 nA or even smaller. However, the photocurrent of the treated silicon sample is in the range from 19 to 21 nA, indicating an 25~30% improvement in the photocurrent. There are several factors affecting the internal quantum efficiency (IQE) of a cell based on black silicon. According to M. Halbwax, the IQE performance of laser-textured solar cells is not only limited by the non-optimized reflection, but also by the surface recombination [21]. And the latter becomes significant because of the large surface. This phenomenon also exists in other reported papers [22, 23]. As shown in Fig. 3, according to Hao-Chih Yuan, the results demonstrated by the IQE was effected significantly by etching times [24]. They fabricated solar cells based on one-sided black silicon wafers and double-side polished planar Si wafers, respectively. Then, the practical standard processes were used to produce a phosphorous diffused front side emitter and an aluminum-back surface field (Al-BSF). With the increased etching time of black silicon, the IQE decreases significantly at short wavelengths. This phenomenon is mainly owing to the high-doping effect and surface recombination mechanism existed in the nanostructured density-graded surface layer.

Hao-Chih Yuan also compared the IQE and reflectivity of the black Si and untreated planar cells [24]. As shown in Fig. 3c, the reflectivity still is below 5% from 350 to 1000 nm after removing PSG after POCl_3_ diffusion and finishing thermal oxidation of the nanoporous layer [25]. The IQE results reveal the main problem in improving the high efficiency of black silicon solar cells that is the remarkable reduction of IQE which exists at short wavelengths. The reduction could be attributed to an inadequate surface passivation present in the front surface of nanoporous layer. The photocurrent and photocurrent density of black silicon-based solar cells are greatly enhanced compared with traditional silicon solar cells. Hao-Chih Yuan demonstrated an increase of greater than 35% in short-circuit current density (*J*_sc_) and conversion efficiency of 16.8% over a planar Si solar cells without anti-reflection [24]. As shown in Fig. 3d, Sanjay K. Srivastava also fabricated black silicon solar cells with the type of n^+^-p-p^+^ structure and compared the performances of silicon nanowire arrays (SiNW-based black silicon) with conventional control solar cells [25, 26].

T. Sarnet fabricated photovoltaic cells with black silicon [27]. The substrates they used are n-type silicon-doped phosphorus to 10^15^ cm^− 3^ (5–20 Ω·cm) and diffused with phosphorus from a POCl_3_ source. The diffused back-side is an n^+^ layer, which could be helpful to form a back ohmic contact between the structured area and the substrate interface. After treating with fs laser, boron dopants were implanted into the front surface by plasma immersion (BF_3_) source and then followed by RTA annealing. With nanosurface structure and p^+^/n/n^+^ device structure, the optical absorption reaches 96%, and the photocurrent has achieved an enhancement of 40% using the laser treatment followed by traditional doping technique. The enhancement of photocurrent is up to 60% when the devices are fabricated by connecting the laser treatment with the plasma ion immersion technique in the photovoltaic cells.

Lu Hu and Gang Chen simulated the optical absorption for the model of periodic nanowire structures [28]. The results from calculation show that the Maxwell-Garnett approach is not suitable for the electromagnetic interaction between each nanowire. The optical absorption in the high-frequency regime can evidently be improved by decreasing the reflection from the nanowire structures. But in the low-frequency regime, no enhancement has been observed owing to the small extinction coefficient of silicon.

Wei Wang et al. proposed a new silicon solar cell design with an embedded metallic nanograting thin film [29]. With a thin metallic nanograting, an enhancement of polarization insensitive absorption could be achieved with a similar absorption at short wavelengths. Erik Garnett and Peidong Yang fabricated the large area silicon nanowires radial p-n junction for photovoltaic device with an efficiency up to 5%, whose short-circuit photocurrents are higher than that of other planar control samples [30]. As there are variations of silicon film thickness and nanowire length, it seems a competition exists between the improved absorption and the increased surface recombination. The results demonstrated when nanowire arrays were made from 8-μm-thick silicon films, the improved absorption could dominate over the increased surface recombination, even without surface passivation. Meanwhile, the micro-structure and surface chemistry of nanoporous black silicon layer techniques have been studied in detail using transmission electron microscopy (TEM) by Yanfa Yan [31]. The results demonstrate that the rough interface of c-Si/suboxide is on the nanometer scale, which also contains a mass of point defects. Fatima Toor et al. fabricated p-type black silicon solar cells with conversion efficiency of 17.1%, and they also analyzed the optical and charge carrier collection performance of multi-scale textured surface [32]. They showed that the spectral response at short wavelengths would be improved as the thickness of nanostructured silicon was reduced. While the nanostructured layer thickness are reduced by 60%, the averaged reflectance of black silicon in solar cell spectrum retains less than 2%. And the spectral response was improved from 57 to 71% at 450 nm.

Except for the application on solar cells, the photoresponse of black silicon in the region of 1 to 1.2 μm also make it applied as digital night vision, plastic sorting for recycling, and noninvasive blood-chemistry monitoring [33]. *G. Scotti* fabricated a micro fuel cell (MFC) combining the hydrogen fuel and a polymer electrolyte for proton exchange membrane [34]. In this MFC, using an appropriate structure, current collector, flow field, and gas diffusion layer integrated on one chip can be realized with black silicon (etched in highly conductive silicon). Under applied bias voltage of 0.7 V, the MFC exhibits a promising performance: 70 mW/cm^2^ power density and 100 mA cm^2^ current density. The results are comparable to that of other similar monolithic devices reported in the literature.

The properties of black silicon make micro-structured silicon available for wide use in commercial devices, not only in solar cells, infrared photodetectors, but also in chemical and biological sensors, as well as field emission devices.

### Field Emission

The rapidly growing area of field emission devices drives the researches to find unique emitting materials, which are required to be robust, easy to fabricate and more favorable emission. Due to the low cost and rich content, the use of silicon devices as emitters are more attractive and available.

In addition to its satisfying optical properties, the micro-structured silicon also exhibits significant field emission characteristics. James E. Carey reported the potential use of black silicon structures in field emission displays, ion thruster propulsion, and microwave amplification [35]. The black silicon structure as emitter shows the low turn-on fields and high current yields, which are important parameters of field emission devices. The relationship between current and voltage for describing potential differences is shown in Fig. 3e [36]. Analysis of the arrays shows that the high, stable field is 1.3 V/μm. Meanwhile, this potential differences can create an emission current density of 1 nA/mm^2^. With fs laser irradiation black silicon, they obtained emission currents up to 0.5 mA/mm^2^ under an applied field of 50 V/μm. The result also demonstrates the low turn-on field and high current yield of black silicon. As shown in Fig. 3f, according to Patrick G. Maloney, as the micro-structure of black silicon changes with the annealing temperature, the emissivity of black silicon also decreases [37].

**Fig. 4:**
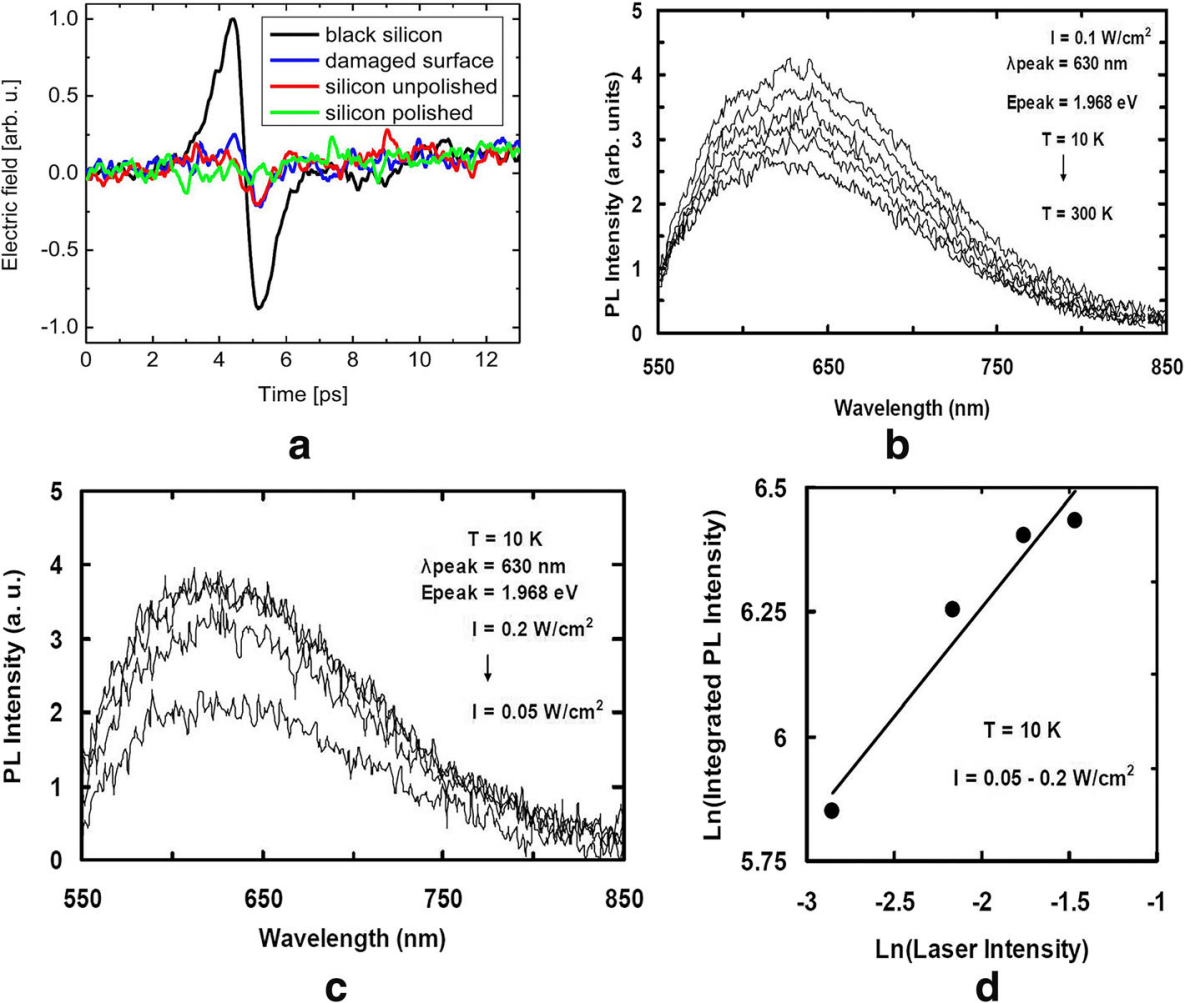
**a** Terahertz electric field for different silicon samples: black silicon, damaged surface, unpolished silicon surface, and polished silicon surface [38]. **b–d** PL spectra of black silicon with different temperature, laser intensity, and wavelength [3]

P. Hoyer reported a study of black silicon as an emitter of terahertz radiation [38]. Due to the structure of black silicon, multiple reflections exist for incident light, leading to an absorption enhancement in nanoscopic needles. The needles are interconnected by the bulk material and confine the charge carriers to separate, which would result in great changes of the local potential differences. Terahertz electric field for different surface qualities is shown in Fig. 4a [38].

### Luminescence

X. Li achieved intense luminescence with porous silicon (PSi) with nanoparticle Au/Pt (deposited on silicon temples with a thin layer (*d* < 10 nm)) fabricated by assisted chemical etching (EtOH:HF(49%):H_2_O_2_(30%) = 1:1:1) in solution of HF and H_2_O_2_ [39]. The results demonstrated that PSi device modified by Pt yields the fastest etching rate and produces the most intense luminescence than that modified by Au. Ali Serpengüzel also reported the photoluminescence (PL) of black silicon samples fabricated by a series of intense and short laser pulses in air [3]. The micro-structured surface of irradiated layer is covered with dendritic nanostructures in the size range from 10 to 100 nm, which will disappear after thermal annealing. As shown in Fig. 4b, the PL spectra of the black silicon measured in the visible light and different annealing temperature excited by a constant laser intensity of 0.1 W cm^− 2^ [3]. And the laser intensity is increased as the PL intensity increases, as displayed in Fig. 4c.

C. Wu observes photoluminescence from SiO_*x*_ formed by laser-structured silicon surfaces in air [38]. PL spectrum just appears at reverse with wavelength (peak value is reached at long wavelength.). As shown in Fig. 4d, the PL intensity increases sub-linearly (i.e., *γ* = 0.44) as the excitation laser intensity enlarges. Generally speaking, the value of coefficient *γ* ranges from 1 to 2 for exciton emission as the excitation energies of photon laser exceed the bandgap of silicon. Moreover, *γ* ≤ 1 stands for being free-bound or bound-bound state recombination. There are bound-bound emission in black silicon through band-tail states recombination for the evidence of *γ* = 0.44 at 10 K.

The relationship between PL intensity and different wavelengths at 10 K is plotted in the Fig. 4c. The results are almost the same as Ali Serpengüzel’s previous study: the variation of photoluminescence with respect to different temperatures in black silicon, as shown in Fig. 4b [3]. The PL intensity decreases as the temperature increases, indicating that a quenching process occurs via radiative recombination (reflected by the large-rate decrease of PL intensity above 120 K). The thermal quenching process, which occurs in the band-tail states (such as impurities and structural defects) of black silicon, has a relationship to the mobility.

G. Kurumurthy also studied the photoluminescence of silicon nanoparticles, fabricated by laser irradiation [40]. The variation of particle size is owing to the irradiation wavelength. They exposed the freshly prepared silicon nanoparticles to air for few days, then observed the PL intensity enhanced and saturated. For the case of exposure to air within 1 h, the PL spectrum of the freshly fabricated samples exhibits two well resolved peaks of ~ 435 and 441 nm, even the measurement is undergoing the constant exposure to the emission of broadening spectrum.

### Surface-Enhanced Raman Spectra (SERS)

**Fig. 5:**
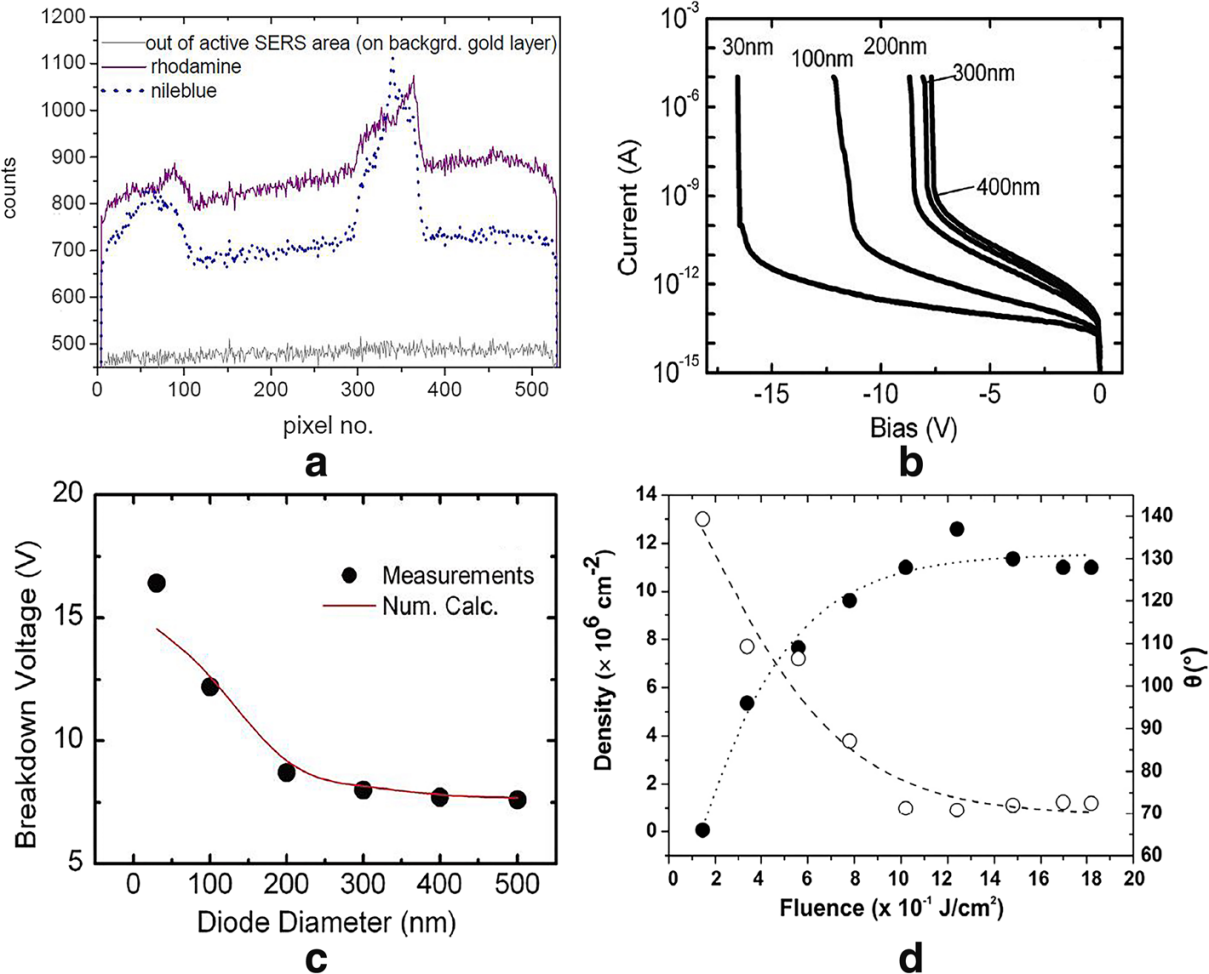
**a** The SERS spectra recorded at low resolution. **b** The current-voltage characteristics at reverse bias for the various diameters of diodes. **c** The measured (dots) and calculated (lines) breakdown voltages of different nanowire diameters. **d** Spikes’ density (empty dots) and the static water contact angle *θ* (full dots) versus fs laser irradiation fluence

Jorg Hubner fabricated an integrated spectrometer device by using epoxy resist (SU-8) on black silicon as Raman spectroscopy and coupling a charge-coupled device (CCD) element [41]. They prepared the black silicon with two methods: (1) an aqueous suspension of gold nanoparticles and polystyrene beads was used to deposit a gold layer on silicon surface, and (2) gold ion were used as the coated catalyst to create the random silicon nanostructures. By using the on-chip spectrometer, they have recorded surface-enhanced Raman spectra of Nileblue and Rhodamin 6G, respectively. As shown in Fig. 5a, the surface Raman spectra recorded by an on-chip spectrometer show that the black silicon integrated system is suitable for Raman sensors. They are low cost and possible to be applied in security monitoring and other “point of care” devices.

### A Hydrophobic Surface

The silicon surface, structured on the micrometer and nanometer scale by fs laser irradiation, exhibited the evident hydrophobic property, as reported by V Zorba [42]. The wettability properties are controlled by a systematic and reproducible variation of the surface roughness, namely the construction of geometrical structure [43]. They varied the laser fluence to obtain the particular hydrophobic surface. Such behavior is called heterogeneous wettability, replaced with another way that air is partially trapped underneath the liquid, which is incomplete penetration within the silicon features. As shown in Fig. 5d, the contact angle of black silicon water increased from 66°to 130°or larger [42].

Later Jia Zhu fabricated the black silicon-based nanodome solar cells with self-cleaning function [44]. The phenomenon is similar to the lotus leaf, which consists of micro-structures and randomly distributed branch-like nanostructures [45]. They modified the black silicon surface with hydrophobic molecules; thus, the nanodome solar cells obtained the self-cleaning function via superhydrophobicity due to the particular morphology. Once black silicon materials are used on solar cells or photovoltaic detectors, dust particles accumulating on the device architectures will seriously imprison sunlight and eventually, leading to the reduction of device efficiency and device life. The devices with self-cleaning function can easily avoid the abovementioned problem.

## Conclusions

As the rapid development of semiconductor industry, the applications of crystalline silicon are much more intensive. Due to the limits of high reflectivity, wide bandgap and indirect bandgap of crystalline silicon, the emergence of black silicon greatly solves the abovementioned problems. The black silicon, with lower reflectivity, higher absorption at wavelengths from 250 to 2500 nm, and excellent optical and electrical properties, becomes an ideal material in some application devices, such as high-efficiency solar cells, near-infrared detectors, and field emission. However, some technology issues also need to be solved about the black silicon materials applied on the devices, such as production efficiency to an industrial scale. Compared with typical metal-assisted chemical etching, reactive ion etching, and photoelectrochemical etching, laser-irradiated process is relatively slow for fabricating porous or nanostructured black silicon. The production rate can be improved by enlarging pulse power, spot size, or increasing scanning speed. And the material damage induced by laser-irradiated process accompanies a form of defects, which requires to be decreased and removed by anneal. The suitable anneal process is the key to achieve high photoresponse and high material quality of photovoltaic applications. How to make better use of black silicon in a specific device still requires further study.

## Abbreviations

Al-BSF: Aluminum back surface field
APD: Avalanche photodiode
CCD: Charge-coupled device
fs: Femtosecond
IQE: Internal quantum efficiency
MFC: Micro fuel cell
near-IR: Near-infrared
NIR/SWIR: Near/shortwave-infrared
PL: Photoluminescence
PSi: Porous silicon
QE: Quantum efficiency
RIE: Reactive ion etching
RTA: Rapid thermal annealing
SNSPDs: Superconducting nanowire single-photon detectors
